# Oral Administration of *Lactobacillus amylovorus* Alleviates Diarrhea by Restoring Gut Microbiota and SCFAs in Neonatal Goats

**DOI:** 10.3390/ani16040633

**Published:** 2026-02-16

**Authors:** Mudathir Y. Abdulrahman, Nasir A. Ibrahim, Mohamed Osman Abdalrahem Essa, Saber Y. Adam, Raza Mohai Ud Din, Rifat Ullah Jan, Nosiba S. Basher, Mokhtar Rejili, Shaaban S. Elnesr, Ahmed A. Saleh, Hosameldeen Mohamed Husien, Mengzhi Wang

**Affiliations:** 1 College of Animal Science and Technology, Yangzhou University, Yangzhou 225009, China; mudathir@duc.edu.sd (M.Y.A.); saber@duc.edu.sd (S.Y.A.); mh24136@stu.yzu.edu.cn (R.M.U.D.); dh23053@stu.yzu.edu.cn (R.U.J.); elemlak1339@yzu.edu.cn (A.A.S.); 008643@yzu.edu.cn (H.M.H.); 2 Department of Biology, College of Science, Imam Mohammad Ibn Saud Islamic University (IMSIU), Riyadh 11623, Saudi Arabia; nsbasher@imamu.edu.sa (N.S.B.); msrejili@imamu.edu.sa (M.R.); 3 College of Veterinary Medicine, Albutana University, Rufaa 22217, Sudan; dh23054@stu.yzu.edu.cn; 4 College of Veterinary Medicine, Yangzhou University, Yangzhou 225009, China; 5 Department of Poultry Production, Faculty of Agriculture, Fayoum University, Fayoum 63514, Egypt; ssn00@fayoum.edu.eg; 6 Animal and Fish Production Department, Faculty of Agriculture (Al-Shatby), Alexandria University, Alexandria City 11865, Egypt; 7 State Key-Laboratory of Sheep Genetic Improvement and Healthy-Production, Xinjiang Academy of Agricultural Reclamation Sciences, Shihezi 832000, China

**Keywords:** *Lactobacillus amylovorus*, diarrhea, gut microbiota, neonatal goats, SCFAs

## Abstract

Diarrhea in kids is a significant health and economic concern for small-scale ruminant farms. This study aims to investigate the properties of *Lactobacillus amylovorus* as a treatment for kids with diarrhea and its effect on the composition of the gut microbiota. A total of 20 neonatal goats (approximately 2 months old) were divided into three groups: healthy control (HC, *n* = 4), diarrhea (D, *n* = 8), and diarrhea treated with probiotic (DT, *n* = 8). We tracked gut microbial profiles, fecal consistency, short-chain fatty acids (SCFAs), and clinical symptoms. Probiotic-treated kids recovered fully from diarrhea within two weeks, while their untreated counterparts showed signs of clinical deterioration and gradual emaciation. Kids with diarrhea had lower microbial richness, according to alpha diversity analysis, and this was only partially restored after probiotic treatment. The kids with diarrhea had the lowest Shannon, ACE, Simpson, Dominance, Pielou-e, and Chao1 indices compared to the HC group, while the administration of *Lactobacillus amylovorus* significantly (*p* < 0.05) restored their normal enrichment in the DT group compared to the D group. The healthy group had a higher abundance of Verrucomicrobiota, while *Firmicutes* and *Bacteroidota* predominated in all groups. *Bacteroides* and *Akkermansia* predominated in the healthy and treated groups. At the genus level, analysis showed elevated levels of *Escherichia-Shigella* and *UCG-005* in kids with diarrhea. In addition, the concentration of each SCFA in the D group was significantly (*p* < 0.05) lower than in the HC group. This study provides novel evidence that *Lactobacillus amylovorus* administration not only alleviates diarrhea but also uniquely restores the production of key SCFAs—including butyrate, acetate, and propionate—in neonatal goats, a finding not previously reported in this species. The concurrent recovery of microbial diversity and SCFA profiles highlights the dual mechanistic potential of *Lactobacillus amylovorus* as a gut microbiota modulator and metabolic therapeutic in young ruminants. These results lend credence to its potential as a probiotic treatment for small ruminant enteric diseases.

## Simple Summary

In newborn goats, diarrheic kid syndrome is a complex illness marked by systemic dysfunction and abnormalities in metabolism. Our study shows a clear connection between dysbiosis and abnormal blood biochemistry, providing new insights into the involvement of gut and gastric dysbiosis in diarrhea etiology. These findings demonstrate the significant influence of *Lactobacillus amylovorus* treatment on the gut microbial composition and point to its potential for regulating gut health.

## 1. Introduction

The gut microbiota plays a critical role in immunity, digestion, and health maintenance. It enhances immune cell production, hormone balance, and nutritional absorption, and also contributes to the development of physiological systems, such as the brain and the immune system [1]. Moreover, the gut microbiota supports the productivity and functionality of the immune system, shaping the host’s immunity and promoting a balanced and tolerant response to innocuous antigens [2,3]. Additionally, the growth and organ development of kids, especially in the early stages of life, are promoted by the gut microbiota. The GM can also influence hormone balance, nutrient absorption in the GIT, and the development of vital physiological systems [4]. Diarrhea is one of the most significant causes of decreased production rates in livestock animals, particularly in neonates with vulnerable gut microbiota. Specifically, diarrhea leads to a decline in the beneficial bacteria that produce short-chain fatty acids (SCFAs), which provide many benefits to animal health. Thus, there may be inevitable correlations between ruminant diarrhea and alterations in the GM. The precise relationships, evolving traits, and underlying mechanisms are nevertheless unidentified. While the trajectory of gut microbiome maturation with age is well-characterized in healthy kids, less is known about how this developmental process interacts with and is modulated by acute diarrheal infection. Therefore, our study design explicitly controls for and analyzes age as a critical factor to disentangle its effects from those of the disease state. Furthermore, recent research on age-related disturbances in ruminant intestinal microecology stability is limited. Previous studies have shown that diarrhea can harm all species, especially lambs, pigs, and chickens with vulnerable gut microbiota [1,5,6]. Besides increased mortality rates, Wang et al. [1] found various alterations in the intestinal microbiota of diarrheal goats. Probiotics have been defined by the International Scientific Association for Prebiotics and Probiotics (ISAPP) as live microorganisms that confer a health benefit to the host when consumed in sufficient quantities [7]. In addition, microbes produce digestive enzymes that assist hosts in the digestion of nutritional particles that host enzymes are not able to break down completely [8]. Furthermore, probiotic development in the animal gut can decrease the amount of undigested carbohydrates, reducing the risk of diarrhea brought on by disruption of the osmotic gradient [9]. The most popular probiotics that can be used in the treatment of a variety of illnesses are *Lactobacillus* sp., *Bifidobacterium* sp., and *Enterococcus* sp. [10]. A strain of *Lactobacillus salivarius* was previously found to treat intestinal damage in mice brought on by *E. coli* [11]. Previous research has confirmed that berberine helps treat colitis and inflammatory bowel diseases [12] and has antibacterial and antidiarrheal functions [13]. *Lactobacillus amylovorus* has a strong resistance to surviving in the animal digestive system, and its clinical use can lead to an increase in the diversity of the gut microbiota in floppy lambs, help regulate the structure of their gut microbiota, suppress the growth rate of pathogenic microbes, and enhance the growth of probiotics. This suggests that administration of *Lactobacillus amylovorus* to diarrheal lambs can help treat diarrheic kid syndrome, and its impact in inhibiting the growth of pathogenic microbes may reduce the use of antibiotics [14].

Metagenomics analysis has, in recent decades, given a great deal of insight into the taxonomic diversity and functions of gut microbiomes. Nevertheless, additional details of microbiomes may yield knowledge on gut microbial communities and their use in animal husbandry. Some studies have demonstrated that ruminants have the capacity to adapt to new diets and have provided an understanding of how the intestinal microbiome interacts with and supports the well-being of animals [15]. Moreover, microbial communities may play a tremendous role in the digestion and uptake of nutrients; therefore, the effects of direct-fed microbes on the rumen microbiota should be established [16]. We hypothesize that oral administration of *Lactobacillus amylovorus* will alleviate diarrhea in neonatal goats by restoring gut microbial homeostasis and increasing the concentration of beneficial SCFAs. This study investigates the therapeutic properties of *Lactobacillus amylovorus* and its effect on the gut microbiota composition, blood biochemistry, and SCFA profiles in diarrheic kids. To assess the impact of *Lactobacillus amylovorus* on diarrheic kids, we employed metagenomic sequencing to characterize specific taxonomic shifts and functional changes in the gut microbiome, moving beyond general diversity metrics to identify key responsive taxa.

## 2. Materials and Methods

### 2.1. Ethics Statement

The Animal Ethics Care and Use Committee of Yangzhou University provided ethical approval for all the animal experiments (approval date: April 2024, approval code: SCXK [Su] 2021-0013). Most importantly, the highest standards of animal welfare were upheld during the animal experiments. The study was developed with strict supervision and scrutiny to prevent any harm to the animals and to enhance humane treatment in all aspects of the research process.

### 2.2. Animal Grouping and Treatment

In total, 20 kids (approximately 2 months old, weighing 10–15 kg body weight) were randomly selected from a goat farm in Taizhou City, Jiangsu Province. The selection criteria were as follows: healthy kids displaying no clinical signs of disease, with normal appetites, activity levels, and fecal consistency.

In addition, diarrheic kids were identified by the presence of loose or watery feces for at least 2 days in a row, accompanied by decreased activity or loss of appetite. All kids were kept in a controlled house with sufficient ventilation, bedding, free access to clean water, and a standard diet (Table 1). The kids were randomly divided into three groups as shown below:

HC: healthy control group (*n* = 4), D: diarrhea group (*n* = 8), and DT: diarrhea + probiotic treatment group (*n* = 8).

Kids were orally fed with the *Lactobacillus amylovorus* suspension at a dose of 5 mL/10 kg of body weight daily. The dosage was adjusted for individual body weight. Blood samples were collected from *n* = 4 to 8 kids per group for analysis.

The suspension was prepared at a concentration of 1 × 10^9^ CFU/mL. In addition, rectal samples were taken to determine their microbiota at baseline.

### 2.3. Blood Biochemical Analysis

Kids with diarrhea exhibited common symptoms, such as an unsteady gait and limb weakness. The blood samples of 2–3 mL were drawn from the kids’ jugular vein using non-anticoagulant vacuum tubes. The blood samples were left to clot at room temperature (for 30 min) and centrifuged at 3000 rpm in order to extract the serum. The serum was then placed into 1.5 mL centrifuge tubes.

### 2.4. Biochemical Index Measurement

An automated biochemical analyzer (Beckman Coulter International Trading Co., Ltd., Shanghai, China) was used to measure 13 biochemical indices, including total protein, albumin, globulin, urea, calcium, creatinine, triglycerides, glucose, total cholesterol, and alkaline phosphatase.

### 2.5. Gut Microbiota Analysis

Fecal samples were collected from a subset of animals to ensure high-quality sampling and reliable data. The selection criteria aimed to guarantee representative samples in both groups, consistent with the inclusion criteria stipulated in Section 2.2.1 [17]. The criteria for the selection process were as follows: (1) Health Status: Only healthy kids without any clinical manifestations of a disease were selected for the healthy control group (as stated in Section 2.2). (2) Consistency between Groups: The kids were selected in such a way that they satisfied the inclusion criteria of each group. In the healthy control group, the healthy + *Lactobacillus amylovorus* treatment group, and the diarrhea *+ Lactobacillus amylovorus* group, the proportion of the neonates was balanced. (3) Non-Risk to Future Collection of Samples: Once a kid had been chosen in a collection, it continued to be in the same collection throughout the study, which ensured uniformity in the sampling procedure. Sterile cotton swabs were used to collect fresh rectal samples of the selected kids (after one kid was selected, no change was possible in further rectal swab collection).

The DNA extraction method used by Li et al. [18] was followed, with 30 ng of extracted DNA used for PCR. Common primers targeting the V3/V4 regions are sensitive to marine archaea and intestinal bacteria, respectively, and are frequently employed for microbial investigation [19,20]. For this reason, PCR was performed using primers *341F* (CCTACGGGNGGCWGCAG) and *806R5* (GGACTACHVGGGTATCTAAT). Using the QuantiFluor™-ST fluorescence quantification system (Promega, San Luis Obispo, CA, USA), the concentration of DNA extracted from 2% gel sugar extraction using the GENEWIZ kit (Guangzhou Genedenovo Biotechnology Co., Ltd., Guangzhou, China) was ascertained. Construction of the PE amplicon library was carried out using the Illumina MiSeq platform in Shanghai, China.

### 2.6. Determination of SCFAs Content

The fecal samples of kids were subjected to liquid chromatography to identify the relative concentrations of the SCFAs, including propionic acid, isovaleric acid, butyric acid, valeric acid, acetic acid, and isobutyric acid after the provision of EcN. Moreover, approximately 50 mg of fresh feces was mixed with 0.5 mL of water, and ultrasonic agitation was performed for 10 min to facilitate preferential extraction. As per the general protocol, centrifugation was performed for 5 min at 4 °C at 12,000 revolutions per minute (rpm). Next, 50 μL of the supernatant was taken and combined with 50 μL of isotopically labeled internal standard (5 μg/mL). Additionally, 50 μL each of 3-nitrophenylhydrazine (3-NPH) solution (250 mM, prepared in 50% methanol/water solution) and 1-ethyl-3-(3-dimethylaminopropyl) carbodiimide (EDC) solution (150 mM, prepared in 75% methanol/water solution with 7.5% pyridine) were also added. Then, the mixture was shaken and left at 30 °C for 30 min. Next, 50 μL of butylated hydroxytoluene (BHT) methanol solution (2 mg/mL) and 250 μL of 75% methanol/water solution were added. The centrifuge tube was subsequently subjected to low-temperature centrifugation for 5 min at 12,000 rpm at 40 °C. After that, 100 μL of the supernatant was collected and placed in an injection vial for detection via mass spectrophotometry. A Waters UPLC BEH C8 column (100 mm × 2.5 mm × 1.7 mm) was used.

### 2.7. Determination of Antioxidant Content

The neonate’s feces were evaluated to measure levels of antioxidant stress markers and related enzymes. We measured the following parameters: malondialdehyde (MDA), superoxide dismutase (SOD), catalase activity (CAT), and adenosine triphosphate (ATP). All analyses were performed using a PU 8720 UV/VIS scanning spectrophotometer. Commercial kits from Nanjing Jiancheng Bioengineering Institute, Nanjing, China, were used for each parameter, and all experimental procedures were conducted according to the manufacturer’s protocols.

### 2.8. Statistical Analysis

In order to refine sequences and build an abundance table of amplicon sequence variants, the raw data of kids were analyzed through Cutadapt and QIIME2 (QIIME 2 2020.6) software [21,22]. ASVs were annotated by blasting against the SILVA database (V138). The Shannon, Simpson, ACE, and Chao1 indices, along with Good’s coverage, were computed in order to assess alpha diversity and investigate the richness and diversity of microflora in the samples [23]. To assess beta diversity, the gut microbiota structure of different kids’ groups was compared using principal component analysis [24], principal coordinates analysis [25], and non-metric multidimensional scaling [26]. Using repeated LEfSe, significantly different microbial species were identified among the groups [27].

The statistical analysis was performed using IBM SPSS Statistics 26. The normality of data distribution was assessed using the Shapiro–Wilk test. For parameters meeting assumptions of normality and homogeneity of variance, one-way analysis of variance (ANOVA) was used to assess the effects of treatment, followed by Tukey’s post hoc test for specific group comparisons. For non-normally distributed data (including alpha diversity indices and some microbial abundance data), the Kruskal–Wallis test was used, followed by Dunn’s post hoc test. A *p*-value < 0.05 was considered statistically significant.

## 3. Results

### 3.1. Probiotic Effects of Lactobacillus amylovorus on Kids’ Health

In the diarrhea group, eight kids received *Lactobacillus amylovorus* orally. All of them stopped having diarrhea after 5 days. In the untreated diarrhea group (D), kids that did not recover spontaneously exhibited progressive emaciation, with fur around the anus filled with feces, weakness of the legs, and a sunken rib cage; they were also frequently lying around the enclosure.

### 3.2. Effects of Lactobacillus amylovorus on Blood Biochemical Parameters

To evaluate the systemic physiological impact of *Lactobacillus amylovorus* supplementation on neonatal goats with diarrhea, we analyzed a comprehensive panel of blood biochemical parameters (Table 2). The results revealed significant alterations in several key markers associated with metabolic and inflammatory status. Alkaline phosphatase activity was significantly elevated across all experimental groups compared to the normal range, with the DT group showing the highest levels (*p* < 0.001). This elevation is consistent with the physiological stress response in diarrheic animals. Total protein and albumin levels, which are critical indicators of nutritional status and liver function, were significantly reduced in the D group compared to the HC group (*p* < 0.01). Notably, *Lactobacillus amylovorus* treatment restored these parameters to levels comparable to those of the healthy controls, indicating improved protein metabolism and nutritional recovery.

The globulin fraction, which reflects immune function and inflammatory status, also showed significant changes. The D group exhibited lower globulin levels compared to the HC group (*p* < 0.05), suggesting impaired immune response during diarrhea. The DT group showed intermediate values, indicating partial restoration of globulin levels following probiotic intervention. Urea levels, a marker of protein catabolism and renal function, were elevated in the D group compared to the HC group (*p* < 0.05), potentially reflecting increased protein breakdown or dehydration. The DT group showed a trend toward normalization, though this did not reach statistical significance compared to the D group.

Other parameters, including glucose, total cholesterol, triglycerides, and calcium, showed no significant differences among the three groups, indicating that these metabolic pathways were less affected by diarrhea or the probiotic treatment under the current experimental conditions.

In summary, the blood biochemical analysis demonstrates that neonatal diarrhea significantly impairs protein metabolism and immune function, as evidenced by reduced albumin, total protein, and globulin levels. Oral administration of *Lactobacillus amylovorus* effectively restored these parameters toward normal levels, suggesting that this probiotic strain contributes to the alleviation of diarrhea by improving nutritional status and supporting immune function in neonatal goats.

### 3.3. Sequence Analysis

**Figure 1:**
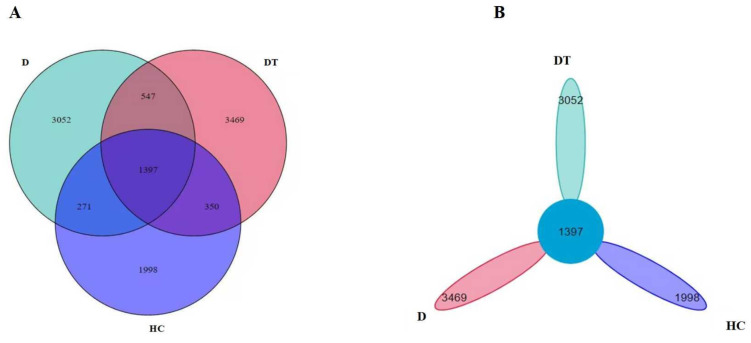
Analysis of gut microbial community features among healthy control (HC), diarrhea (D), and diarrhea-treated (DT) kids. (**A**,**B**) Venn diagrams showing the number of unique and shared operational taxonomic units (OTUs) between groups.

The Illumina HiSeq 2500 platform generated 1,078,215 paired-end reads, with an average of 53,911 per sample. All samples yielded 995.723 high-quality readings after quality control processing and the removal of unqualified data; the average number of reads per sample ranged from 98.232 to 10.373 (Supplementary File S1, Table S1). In taxonomic assignment, a total of 8519 OTUs were identified from all samples, and 271 common OTUs were discovered, accounting for approximately 4.39% of the total OTUs, according to the DEBLUR algorithm. In particular, the FA, KA, and LA included 3469, 3052, and 1998 OTUs, respectively, while the common OTUs totaled 1397 (Figure 1A,B and Supplementary File S1).

**Figure 2:**
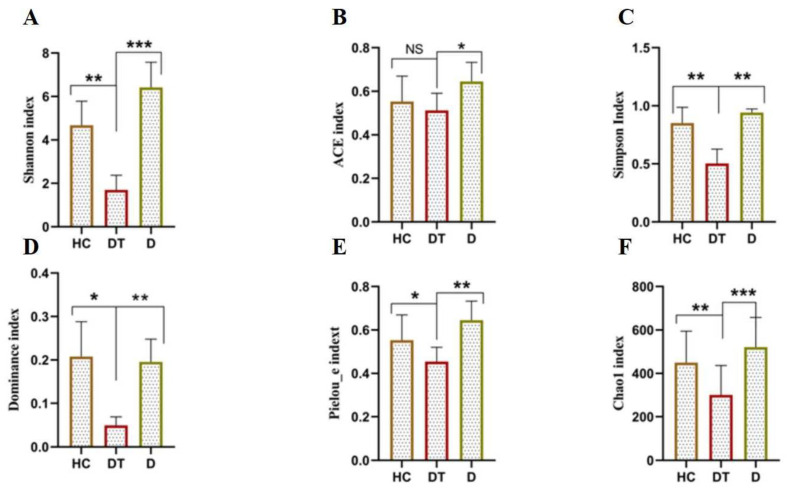
Alpha diversity indices: Shannon (**A**), ACE (**B**), Simpson (**C**), Dominance (**D**), Pielou’s evenness (**E**), and Chao1 (**F**). Significant differences between groups are indicated with brackets and asterisks (* *p* < 0.05, ** *p* < 0.01, *** *p* < 0.001). Comparisons shown are between the HC and D groups and between the D and DT groups.

**Figure 3:**
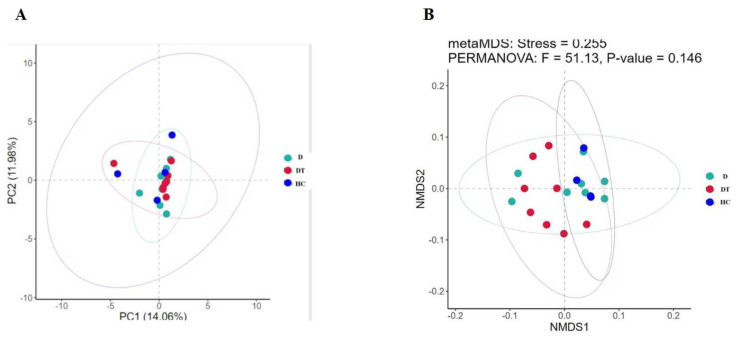
(**A**,**B**) Principal coordinate analysis (PCoA) plots illustrating beta diversity based on Bray–Curtis distances.

To determine the difference between the diversity of the intestinal microbes in the three groups, alpha and beta indices were assessed using high-quality sequences to analyze variations in the intestinal microbial communities. The alpha diversity of the gut microbiota was calculated through the diversity indices (Simpson) and community abundance (Chao1, Shannon, Dominance, Pielou-e, and ACE). The kids displaying diarrhea had the lowest Shannon, ACE, Simpson, Dominance, Pielou-e, and Chao1 indices compared to the HC group, while the administration of *Lactobacillus amylovorus* significantly (*p* < 0.05) restored these indices in the DT compared to the D group (Figure 2A–F). In addition, the beta diversity analysis revealed that the samples in the DT, D, and HC groups were grouped together (Figure 3A and B).

### 3.4. Bacterial Community Composition at Different Taxonomic Levels

**Figure 4:**
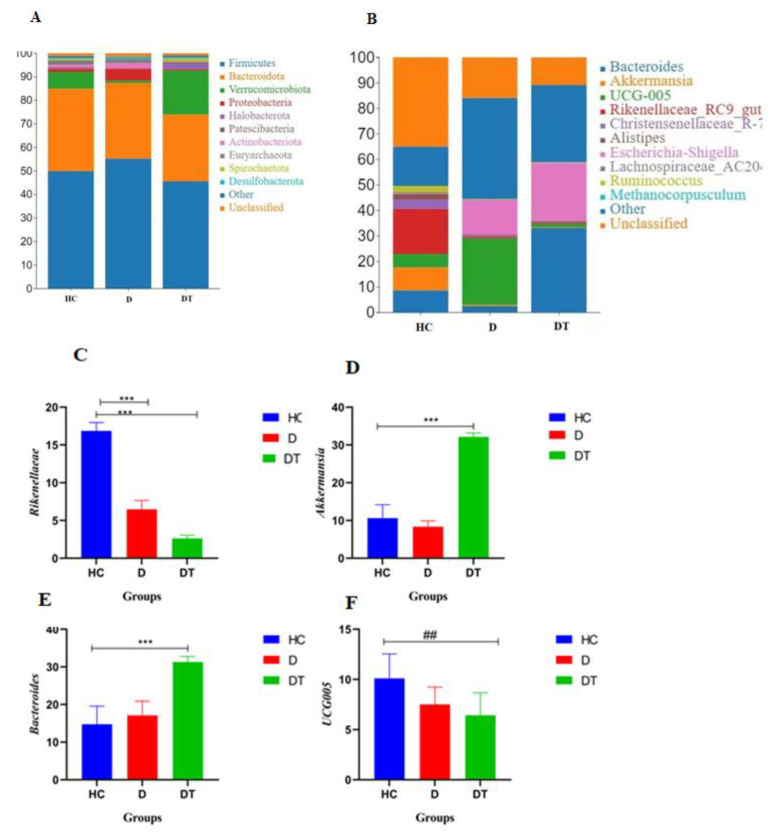
Relative abundance of the top 10 most prevalent gut microbial taxa at both phylum and genus levels among the three groups. Panels (**A**) and (**B**) depict the relative abundance of gut microbiota in each sample at the phylum and genus levels, respectively. The abundance of the top four genera in the small goat fecal microbiota of each group: (**C**) *Akkermansia*, (**D**) *Bacteroides*, (**E**) *Rikenellaceae-RC9-gut-group,* and (**F**) UCG 005. Significant differences between groups are indicated with brackets and asterisks (*** *p* < 0.001, ^##^ *p* < 0.01). Comparisons shown are between the HC and D groups and between the D and DT groups.

Microbial taxonomic assignment in the DT, D, and HC groups was used to determine the dominant phyla and genera (Figure 4). *Firmicutes* (54.23%, 47.60%, and 44.58%) and *Bacteroidota* (55.64%, 43.08%, and 40.24%) were the most prevalent phyla in all three groups, making up around 97% of the taxonomic groupings found (Figure 4A). In contrast, the phylum *Verrucomicrobiota* in the D group was less abundant (34.67%), differing from the other groups. Other phyla, such as *Proteobacteria*, *Halobacterota*, *Patescibacteria*, *Actinobacteriota*, *Euryrchaeota*, *Spirochaetota*, and *Desulfobacterota*, were represented with a lower abundance (Table S2). At the genus level, the most common bacteria in the HC and D groups were *Bacteroides* (55.09%) and *Akkermansia* (51.69%), which collectively accounted for 87% and 93% of the total bacterial composition, respectively (Figure 4B). *Bacteroides* was reduced in the DT group.

**Figure 5:**
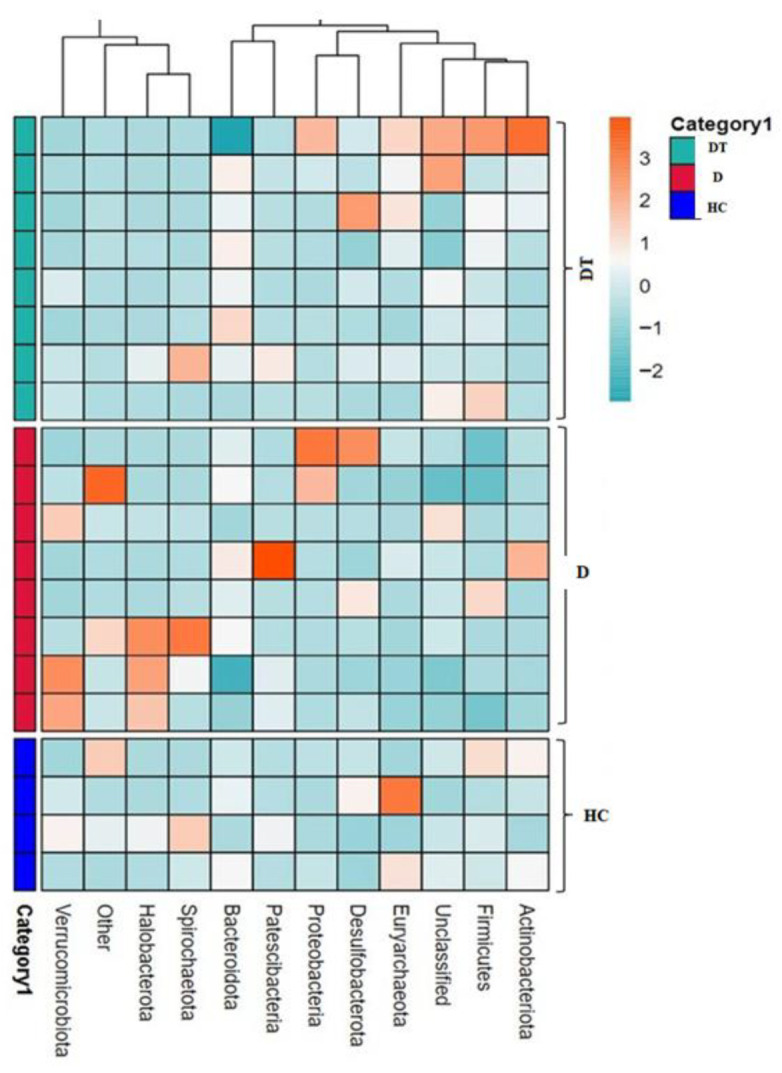
Heatmap of the relative abundance of bacterial communities at the genus level in each sample. Each colored block in the heatmap represents the relative abundance of a specific bacterial genus within that sample.

Furthermore, the most common bacteria genera in the D and DT groups were *Escherichia* and *Shigella* (33.90% and 27.75%), while UCG-005 (23.35%) was found to be higher in the DT group and lower in the other two groups. Other genera were present at lower levels across all groups (Table S3). The relative richness of these microorganisms is also demonstrated in a heatmap generated via clustering analysis (Figure 5).

### 3.5. LEfSe Analysis

**Figure 6:**
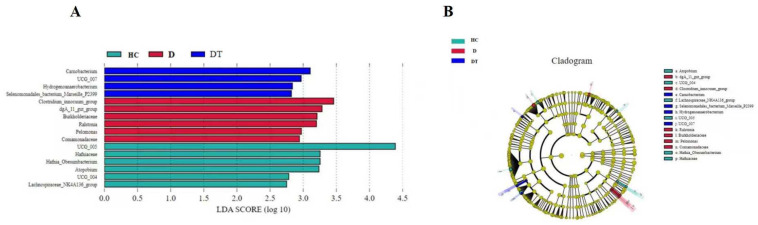
LEfSe analysis for notable alterations in the differential fecal microbiota in all groups. (**A**,**B**) in the three groups, the biomarker bacterial species were identified by LEfSe analysis (LDA score > 4).

Linear discriminant analysis effect size (LEfSe), in combination with linear discriminant analysis (LDA), was performed at different levels of classification to compare differences in intestinal microbiota among the four groups. The findings showed that the HC, D, and DT groups contained four, six, and six distinct bacterial taxa, respectively (Figure 6A,B). The addition of Lactobacillus bacteria to the DT group clearly had a significant impact on the composition of microbes at every level, from phylum to genus. *Cambacterium*, *UCG_007*, *Hydroganoerobacterium*, *and Serlenomonadalus bacterium_marscilla-P2399* were found in high abundance in the HC group. The *Clostridium innocuum* group, *Burkholderiaceae*, *Ralstonia*, *Pelomonas*, *and Comamonadaceae* were more abundant in the D group, while the relative abundance of *UCG005*, *Hafniaceae*, *Hanfnia_Obesnmbacterium*, *Atopobium*, *UCG_004*, and *Lachnosdpiraceae NK4A1346_*group was considerably higher in the FA group.

### 3.6. Lactobacillus amylovorus Increases Content of SCFAs

**Figure 7:**
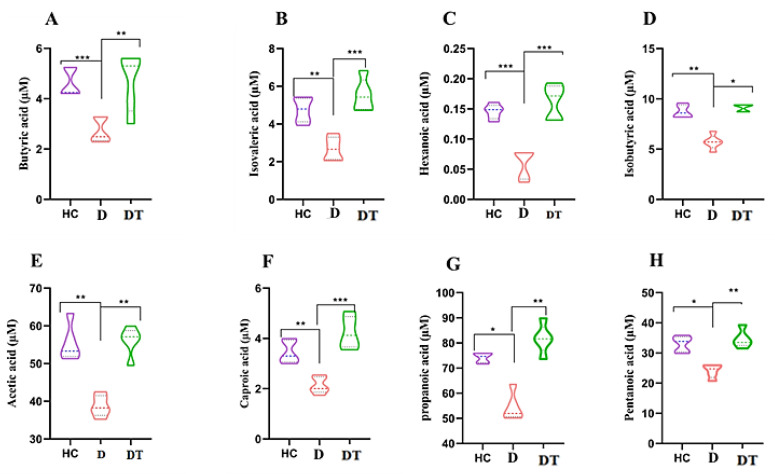
Concentrations of SCFAs in fecal samples from HC, D, and DT kids. (**A**) Butyrate; (**B**) Isovalerate; (**C**) Caproate; (**D**) Isobutyrate; (**E**) Acetate; (**F**) Valerate; (**G**) Propionate; (**H**) Heptanoate. Data are presented as mean ± SD. Significant differences between specific groups are indicated with brackets and asterisks (* *p* < 0.05, ** *p* < 0.01, *** *p* < 0.001; one-way ANOVA with Tukey’s post hoc test). For each SCFA, comparisons were made between the HC and D groups and between the D and DT groups.

The composition of butyric acid, isobutyric acid, hexanoic acid, isovaleric acid, acetic acid, caproic acid, propanoic acid, and pentanoic acid was identified, and the profiles are indicated in Figure 5. Each of the SCFAs in the D group was significantly (*p* < 0.05) lower than in the HC group of kids. *Lactobacillus amylovorus* administration in the diarrhea group significantly increased the levels of butyric acid, isobutyric acid, hexanoic acid, isovaleric acid, acetic acid, caproic acid, propanoic acid, and pentanoic acid (*p* < 0.05) compared to the D group (Figure 7A–H).

### 3.7. Effects of Lactobacillus amylovorus on Antioxidant Enzymes and Oxidative Stress

**Figure 8:**
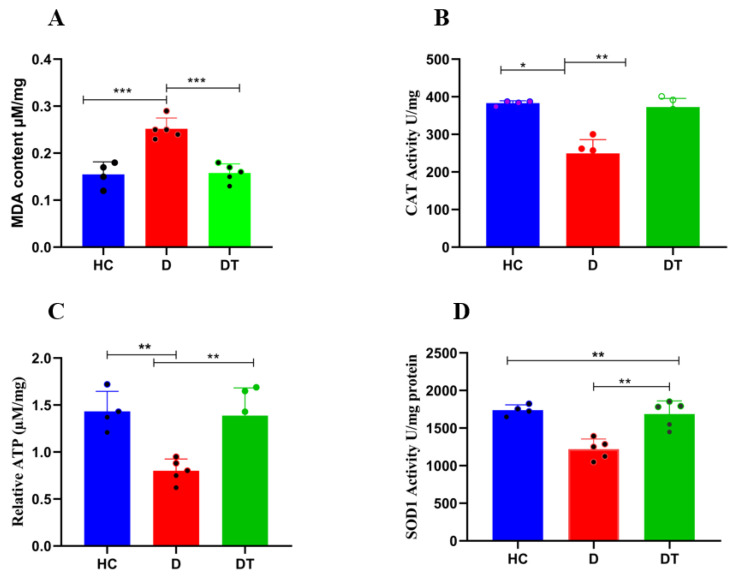
Effect of *Lactobacillus amylovorus* on oxidative stress and antioxidant parameters in goat kids. (**A**) MDA, (**B**) CAT, (**C**) ATP, (**D**) SOD. Data are presented as mean ± SD. Significant differences between specific groups are indicated with brackets and asterisks (* *p* < 0.05, ** *p* < 0.01, *** p < 0.001; one-way ANOVA with Tukey’s post hoc test). For each parameter, statistical comparisons were performed between the HC and D groups and between the D and DT groups.

The contents of MDA, SOD and CAT were determined, and the results are shown in Figure 8. The result showed significant differences between the diarrheal group (D) and the control group (HC). Diarrhea increased oxidative stress when compared with the control (HC) and treated group (DT), but there was no significant difference between the treated group (DT) and the control group (HC), indicating that *Lactobacillus amylovorus* decreased the oxidative stress (Figure 8A). Additionally, the antioxidant activities of SOD and CAT were significantly higher in the *Lactobacillus amylovorus-*treated group (DT) compared to the diarrheal group (D), but there was no significant difference between the *Lactobacillus amylovorus*-treated group (DT) and the control group (HC) (Figure 8B–D). Furthermore, ATP production was significantly higher in the *Lactobacillus amylovorus-*treated group (DT) compared to the (D) group. However, there was no significant difference in the *Lactobacillus amylovorus-*treated group (DT) compared to the control group (HC) (Figure 8C).

### 3.8. Correlation Network Analysis of Microbiota–Host Interactions

**Figure 9:**
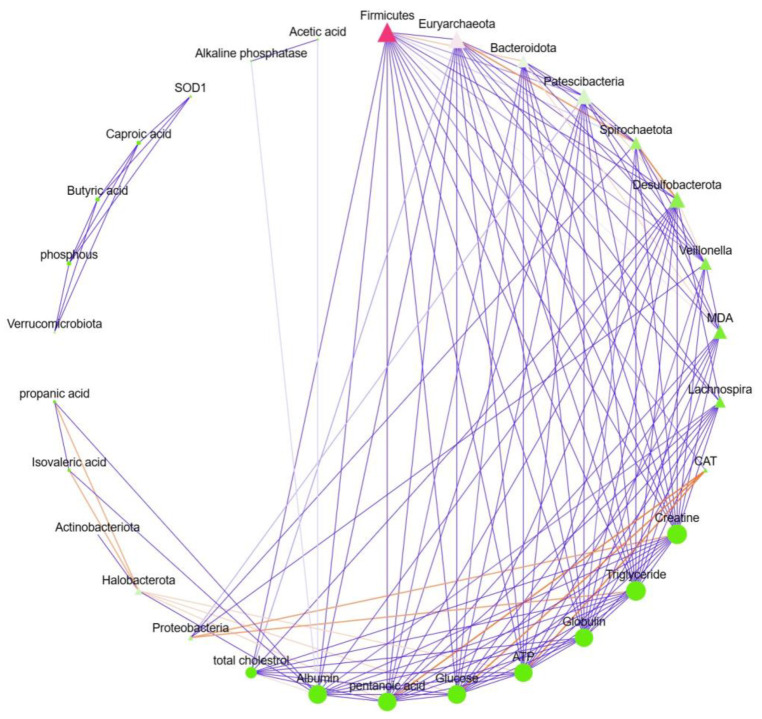
Correlation network between gut microbiota and host phenotypic parameters. Network analysis showing significant Spearman correlations between dominant microbial taxa (nodes in purple) and key host phenotypic variables (nodes in orange). Red edges represent negative correlations, while green edges represent positive correlations. The size of each node is proportional to the number of connections (degree). Microbial taxa are labeled at the phylum level (e.g., *Firmicutes*, *Bacteroidota*) or genus level, where specified. Phenotypic variables include blood biochemical parameters (e.g., alkaline phosphatase and globulin), antioxidant markers (SOD1, CAT, and MDA), and metabolic compounds (e.g., acetic acid). The network illustrates potential mechanistic links between microbiota remodeling and host physiological recovery following *Lactobacillus amylovorus* administration.

To elucidate the potential mechanistic links between gut microbiota remodeling and host physiological recovery, we performed a comprehensive correlation analysis between the relative abundances of dominant microbial taxa and key phenotypic parameters (Figure 9). The network revealed multiple significant associations that provide insight into how *Lactobacillus amylovorus*-induced microbial shifts may contribute to diarrhea alleviation.

Beneficial microbial taxa showed strong positive correlations with favorable metabolic and antioxidant markers. The phylum *Firmicutes*, which was enriched in the DT group, exhibited positive associations with acetic acid (a major short-chain fatty acid) and key antioxidant enzymes, including SOD1 and CAT. Conversely, *Firmicutes* showed a negative correlation with MDA, a marker of oxidative stress. This correlation pattern suggests that the restoration of *Firmicutes* abundance following probiotic treatment may contribute to enhanced SCFA production and improved antioxidant capacity, thereby mitigating oxidative damage associated with diarrhea. Conversely, certain microbial taxa were associated with adverse metabolic profiles. The phylum *Bacteroidota* showed positive correlations with triglyceride levels and negative associations with total cholesterol, indicating potential involvement in lipid metabolism dysregulation during diarrhea. Additionally, several microbial nodes exhibited connections with alkaline phosphatase and globulin, both of which were significantly altered in diarrheic animals, suggesting that microbiota–host interactions may influence inflammatory and metabolic stress responses.

The network structure revealed complex interrelationships between microbial communities and host physiology. Multiple microbial taxa were interconnected with several phenotypic parameters, indicating that the beneficial effects of *Lactobacillus amylovorus* are likely mediated through coordinated changes in the gut ecosystem rather than isolated microbial shifts. The presence of hub nodes (e.g., *Firmicutes* and *Bacteroidota*) with multiple connections underscores their central role in modulating host metabolism and redox homeostasis.

In summary, the correlation network analysis provides direct evidence linking specific microbial taxa to host metabolic and antioxidant parameters. These associations support the hypothesis that *Lactobacillus amylovorus* alleviates diarrhea by restoring a microbial community that promotes SCFA production, enhances antioxidant defense, and modulates metabolic homeostasis in neonatal goats.

## 4. Discussion

In newborn goats, diarrheic kid syndrome is a complex illness marked by systemic dysfunction and abnormalities in metabolism. Our study shows a clear connection between dysbiosis and abnormal blood biochemistry, providing new insights into the involvement of gut and gastric dysbiosis in diarrhea etiology. A complex interdependence between the increase in pathogenic bacteria, the depletion of probiotics, and metabolic disturbances is revealed by the metagenomic sequencing and blood biochemical analyses. Goats with the DT group had significantly higher levels of albumin, phosphate, and alkaline phosphatase, according to blood biochemical tests. The substantial increase in alkaline phosphatase suggests that acidosis is specifically linked to the excess proliferation of *Escherichia coli,* which ferments undigested lactose to generate excessive lactic acid and releases toxins like *Staphylococcus aureus* α-hemolysin and *Escherichia coli* lipopolysaccharide that worsen symptoms [28,29,30,31].

Few studies on the variations of the GM in goats of different ages and health statuses have been published, despite the fact that studies on the mammalian intestinal microbiota have so far addressed a wide range of topics, including metabolism, physiology, and immunology [32]. In this study, gut microbial diversity and composition were analyzed in fecal samples of control, healthy, and diarrheal kids. A significant difference in bacterial communities was found between healthy kids treated with *Lactobacillus amylovorus* and diarrheal kids. While the complete depth of microbial diversity is shown by promising studies, inter-individual variations continue to pose a challenge. Metagenomic analysis and larger cohorts may improve the study’s generalizability and yield useful information. Numerous factors, including infectious agents, weaning, and management, have been identified in previous research as potential causes of diarrhea in cows. The majority of these parameters, as well as dietary, physiological, and environmental stressors [33,34], have been connected to abnormalities of normal intestinal flora because they are crucial for the intestinal function of animals. Microbiome analysis has been employed in several recent studies to determine the gut microbiota of diarrheic animals, with a particular focus on lambs [35], commercial piglets [36], early-weaned Tibetan piglets [37,38], neonatal dairy calves [39,40], and suckling goats [1]. Han et al. demonstrated that diarrhea may be mechanistically related to gut microbial dysbiosis [6]. In the current study, the microbial composition of samples from different groups showed significant variations, indicating that despite these differences, there was minimal overall variation in gut microbial community structure. The administered dose of *Lactobacillus* modulates the microbial state of equilibrium, which lowers alpha diversity in the DT group. The HC group’s partial recovery of diversity measures, however, points to a modulatory effect that might enhance some taxa without significantly changing the overall microbial architecture.

At the phylum level, probiotic intervention dramatically changes the gut microbiota, with *Firmicutes* and *Bacteroidota* predominating the bacterial population, suggesting possible connections between probiotic intervention and diarrhea. This is consistent with previous studies that identified *Firmicutes*, *Bacteroidota*, and *Pseudomonadota* as the dominating phyla in the intestine of Hainan black goats. Fascinatingly, Hainan black goats have a greater intestinal content of *Bacteroidota* (apart from the ileum) than Yimeng black goats [18,41]. However, a disturbance in the microbial balance was indicated by the lower levels of *Bacteroidota* in the diarrhea-infected (D) group and higher levels in the control group (HC). In contrast to the DT and HC groups, the D group had a greater *Verrucomicrobiota*, which may indicate gut inflammation or mucosal injury. Lower amounts of less prevalent phyla, like *Proteobacteria*, were found. The key novel finding of our study is the specific microbial restructuring induced by *L. amylovorus*. Treatment significantly reduced the abundance of potentially pathogenic genera that were elevated in diarrheic kids, such as *Escherichia-Shigella*, while promoting the recovery of beneficial genera like *Akkermansia*, which is crucial for mucin degradation and barrier integrity. Furthermore, the treated group (DT) exhibited a distinct increase in UCG-005, a genus whose role in goat kid health warrants further investigation but may be linked to the observed metabolic recovery. These targeted shifts were directly correlated with the restoration of SCFA profiles.

The presence of dominating genera has a significant impact on gut health; the most prevalent genera are *Bacteroides* and *Akkermansia*, which indicate a balanced microbiota. This result is in line with a recent study that revealed that, in neonates with diarrhea, *Bacteroides* continued to be the most common genus and tended to grow as a typical GM component in ruminant guts [1]. Furthermore, the genus *Escherichia-Shigella* exhibited a significant enrichment in both the DT and HC groups, with the D group displaying especially high enrichment (33.90%).

According to the latest research, the colon has a higher relative abundance of *Bacteroides*. The *Christensenellaceae R-7* group demonstrated the ability to produce acetate and butyric acid from carbohydrates [42]. Studies indicate that a lack of *Christensenellaceae* may contribute to obesity [43]. Despite being a normal part of the flora, *Escherichia coli* strains are often linked to infection, diarrhea, and inflammation of the gut due to their increased abundance and pathogenic variants. According to the most recent research, the main genus groups in the stomach of black goats are *Eubacterium parthenogenesis*, *Christensenellaceae_R-7_group*, *Rikenellaceae_RC9_gut_group*, *Bacteroides*, *Paeniclostridium*, and *Ruminococcaceae_UCG-005*. *Prevotella-1.* The composition of the gut microbiota in goats changes as they age [44,45,46,47]. Although the genus *UCG-005* is associated with disease in the D group, it is unknown how it affects host physiology. Future research should look into how probiotics affect the composition and functionality of microorganisms over the long term.

Key bacterial biomarkers enriched in each experimental group were identified by the LEfSe technique in conjunction with LDA scoring. This revealed distinct microbial signatures that were altered by the probiotic intervention and the disease state. Taxa including *Cambacterium*, *UCG_007*, *Hydrogenoanaerobacterium*, and *Selenomonadales bacterium_marscilla-P2399* were found to be more abundant in the control group (HC). These taxa are typically linked to a healthy, balanced intestinal environment. The idea that a stable microbiota is made up of specialized and varied taxa that support gut health by producing short-chain fatty acids, maintaining mucosal integrity, and being metabolically versatile is supported by their prevalence in the DT group. *Lactobacillus amylovorus* may affect microbial reorganization by boosting commensals or changing the intestinal environment, possibly facilitating gut repair or competitive exclusion of pathogens, as seen by the considerable enrichment in certain taxa displayed by the *Lactobacillus*-treated group.

Our study’s complex microbial connections provide important new information about the gut microbiota’s possible functional interactions and competitive dynamics. A common ecological niche or mutualistic relationship may be indicated by the positive correlation seen between *Akkermansia* and genera like *UCG_099*, *UCG_005*, Complobacter, and *Escherichia/Shigella*. Researchers have also found that adding *Lactobacillus* to a mouse’s diet every day can help prevent non-alcoholic fatty liver by improving gut health and lowering swelling in mice [48]. Adding *Lactobacillus* to a host’s diet also increased digestive enzyme activity and antioxidant capacity, which is good for the host [49,50]. A bacterium that breaks down mucin, *Akkermansia*, is frequently regarded as advantageous for preserving metabolic health and the integrity of the intestinal barrier. *Lactobacillus* supplements, on the other hand, greatly decreased the abundance of *Paenibacillus*, *Aerococcus*, *Comamonas*, and *Corynebacterium* in the ileum of mice with *E. coli* infection. *Paenibacillus* infections are very uncommon, but they can sometimes lead to meningitis [51]. Its favorable association with *Escherichia/Shigella*, which contains recognized pathogens, however, points to a complicated and context-dependent role that is impacted by the host’s diet or immunological condition. The co-occurrence of *UCG* and *Campylobacter* species could be an indication of a cooperative metabolic network that facilitates substrate utilization and mucosal colonization.

In goat kids with diarrhea, these multifaceted functions of SCFAs are critical for modulating the gut environment, host immunity, and pathogen interactions [52]. SCFAs, primarily acetate, propionate, and butyrate, are absorbed by intestinal epithelial cells, providing a vital energy source for the ruminant gut [53,54]. Butyrate is a major energy source for colonocytes and is critical for maintaining intestinal barrier function. Research indicates that SCFAs modulate gene expression related to SCFA transporters and inflammatory responses in goat jejunum epithelial cells, suggesting their direct involvement in maintaining gut homeostasis [54]. SCFAs are products of microbial fermentation, and their levels are intrinsically linked to the composition and metabolic activity of the gut microbiome [55]. In diarrheal conditions, the metabolic activity of the intestinal microflora can influence the severity and duration of illness. Early weaning and milk substitutes in goat kids have an impact on the gut microbiome, metabolomics, and antibodies, affecting the presentation of diarrhea [56]. SCFAs can inhibit the growth and virulence of pathogenic bacteria. They interfere with the replication and virulence of pathogens, such as *E. coli*, by modifying the production of pathogenic factors like toxins and by decreasing the gut pH, which creates an unfavorable environment for many harmful bacteria [57]. This resistance against enteric pathogens is vital in preventing and treating diarrheal diseases. SCFAs contribute to strengthening the intestinal epithelial barrier. They promote mucin production and enhance tight junctions between epithelial cells, which are essential for maintaining gut integrity and reducing the passage of pathogens from the gut into the bloodstream [58].

The localized acidity of the gut due to the generation of lactic acid is a fundamental mechanism behind the advantages of *Lactobacillus amylovorus*. Acid-sensitive pathogens (like *Escherichia-Shigella*) are directly inhibited by this output [59], and it may benefit acid-tolerant commensals that are associated with health, including certain *Lachnospiraceae* and *Akkermansia* [60,61]. Crucially, lactic acid’s dissociated form has the ability to damage bacterial membranes on its own, offering an antibacterial action that goes beyond a reduction in pH [62]. The observed microbial reorganization and recovery of SCFA profiles can be explained by this selective ecological pressure.

A compromised intestinal barrier is a common feature of diarrheal conditions, and SCFA-mediated reinforcement can aid recovery. The recovery of SCFA concentrations—particularly butyrate and acetate—in the DT group provides a direct functional link between the observed microbial shifts and clinical improvement. Butyrate serves as the primary energy source for colonocytes, promoting epithelial repair, while acetate contributes to systemic energy metabolism. The restoration of these key metabolites aligns with the normalization of blood biochemical parameters, such as albumin and total protein, and the resolution of diarrhea, demonstrating a coherent mechanism of action from probiotic administration to microbial modulation, metabolic output, and systemic host recovery.

There are some serious limitations to this investigation. The first limitation is the modest and unbalanced sample size, particularly the small healthy control group *(n* = 4). While the results show clear trends, this imbalance may affect the statistical power of some comparisons, such as alpha diversity indices, and limit the generalizability of the findings. Future studies with larger, balanced cohorts are warranted to confirm these observations. Reproducibility problems like false positives and false negatives may be exacerbated when studying goat kids. Second, fecal samples were collected rather than gut samples to study the gut microbiota of kids who had diarrhea. Conversely, feces could be valuable sample sources for studying diseases and biomarkers in humans and animals, which can provide reliable host data.

## 5. Conclusions

These results demonstrate the significant influence of *Lactobacillus amylovorus* treatment on the gut microbial composition and point to its potential for regulating gut health. The effects of *Lactobacillus amylovorus* are likely mediated not only through direct host interactions but also through its metabolic activity (i.e., acid production), which restructures the gut microbial ecosystem to favor a healthier, SCFA-producing community. These findings support the potential of this probiotic as a beneficial intervention for the management of enteric diseases in small ruminants, contributing to improved animal health and enhanced economic sustainability on small-scale farms.

## Figures and Tables

**Table 1:** Composition of ingredients of goat feed.

Ingredients	%	Nutrition Composition (Per kg)
Corn	50	Crude protein 10%, metabolizable energy 3.3%, starch 65%
Pea skin	20	Fiber 40%, fat 2%, ash 3%
Silage	15	Dry matter 35%, crude protein 9%, calcium 0.3%, phosphorus 0.3%
Alfalfa meal	15	
Soybean meal	50	Amino acid (lysine 3.3%, methionine 0.6%, threonine 1.8%, tryptophan 0.6%
Sunflower meal	50	Crude protein 32%, sulfur 0.3%, magnisum 0.3%)
Iodizal salt	10	NaCl 97%, KI 20%, KIO_3_ 30%
Wild hay powder	50	Fiber 3%, protin 1.2%
Additives	1	Vitamins (A, D, E), trace minerals (Zn, Se)
Bran	320	ME, 4%, fat 4%

**Table 2:** Blood biochemical parameters.

Item	Normal Range	Experiment Groups			*p*-Value
		**HC**	**D**	**DT**	
Alkaline phosphatase (U/L)	93 ≈ 38	265.75 ± 58.66 ^a^	181.5 ± 32.85 ^a^	407.36 ± 36.65 ^a,b^	<0.001
Total protein (g/L)	52 ≈ 82	68.50 ± 7.51 ^a^	61.70 ± 6.65 ^a^	68.30 ± 2.92 ^a^	0.123
Albumin (g/L)	25 ≈ 45	35.24 ± 2.71 ^a^	26.25 ± 2.42 ^b^	36.80 ± 2.50 ^a^	<0.01
Globulin (g/L)	25 ≈ 45	43.05 ± 9.83 ^a^	35.47 ± 7.22 ^b^	41.50 ± 8.90 ^a,b^	<0.05
Albumin:globulin ratio	0.6 ≈ 1.2	0.82± 0.21 ^a^	0.74 ± 0.17 ^a^	0.89 ± 0.19 ^a^	0.234
Urea (mmol/L)	3.6 ≈ 7.1	8.76 ± 2.15 ^a^	11.45 ± 1.39 ^b^	9.10 ± 1.50 ^a^	<0.05
Creatine (μmol/L)	62 ≈ 106	59.75 ± 3.62 ^a^	60.47 ± 5.44 ^a^	58.20 ± 4.10 ^a^	0.234
Glucose (mmol/L)	2.8 ≈ 5.0	5.38 ± 0.36 ^a^	5.77 ± 0.73 ^a^	5.60 ± 0.5 ^a^	0.345
Total cholesterol (mmol/L)	1.3 ≈ 3.9	2.22 ± 0.34 ^a^	2.20 ± 0.30 ^a^	2.35 ± 0.28 ^a^	0.567
Triglyceride (mmol/L)	0.1 ≈ 0.3	0.28 ± 0.06 ^a^	0.26 ± 0.05 ^a^	0.29 ± 0.04 ^a^	0.456
Calcium (mmol/L)	2.2 ≈ 2.8	2.45 ±0.21 ^a^	2.33 ± 0.18 ^a^	2.51 ± 0.15 ^a^	0.112

Different superscript letters (a, b) within a row indicate significant differences (*p* < 0.05).

## Data Availability

The original contributions presented in this study are included in the article/Supplementary Materials. Further inquiries can be directed to the corresponding authors. The 16S rRNA gene sequences analyzed in this study are provided in Supplementary File S1.

## Supplementary Materials

The following supporting information can be downloaded at https://www.mdpi.com/article/10.3390/ani16040633/s1, Table S1: Relative abundance of the top 10 most prevalent gut microbial taxa at phylum; Table S2: Relative abundance of the top 10 most prevalent gut microbial taxa at genus; Table S3: The sequence information of each sample.
