# Supplementary material for: Oral Administration of Lactobacillus amylovorus Alleviates Diarrhea by Restoring Gut Microbiota and SCFAs in Neonatal Goats
Source: Animals (Basel). 2026 Feb 16;16(4):633. doi: 10.3390/ani16040633 (PMC12937376; doi:10.3390/ani16040633)
Supplement: Supplementary file 1 [file animals-16-00633-s001.zip › Supplementary File 1, Table S.pdf]

Table S1. Relative abundance of the top 10 most prevalent gut microbial taxa at phylum

Taxon	LA	FA	KA
Firmicutes	47.28	49.10	45.61
Bacteroidota	36.14	33.27	28.34
Verrucomicrobiota	9.65	11.31	18.80
Proteobacteria	1.00	0.92	0.53
Halobacterota	0.29	0.67	2.31
Patescibacteria	0.30	0.41	0.53
Actinobacteria	1.04	0.32	0.20
Euryarchoeota	1.52	0.47	0.20
S pirochaetata	0.38	1.40	1.24
Desulfobacterota	0.67	0.50	0.52

Table S2. Relative abundance of the top 10 most prevalent gut microbial taxa at genus

Taxon	LA	FA	KA
Bacteroides	12.26	9.80	9.05
Akkermonsia	9.60	11.27	18.72
UCG -005	9.06	7.09	6.42
Rikenellaceae RC9 gut group	7.45	5.74	6.51
Christensenllaceae R7 - group	3.85	2.85	3.83
Alistipes	3.45	3.29	2.01
Escherichia - Shigella	0.86	0.38	0.34
Lachnospiraceae AC2044- group	0.44	1.43	1.05
Methanocrpusulum	0.29	0.67	2.31
Ruminococcus	2.39	1.55	1.00

Table S3. The sequence information of each sample

Sample ID	Raw Reads	Clean Reads	Effective Reads	Effective (%)
FA1	82,246	82,231	66,945	81.4
FA2	58,629	58,616	46,772	79.78
FA3	98,252	98,232	79,489	80.9
FA4	88,269	88,256	70,281	79.62
FA5	10,375	10,373	83,877	80.84
FA6	87,303	87,280	69,420	79.52
FA7	96,347	96,321	77,510	80.45
FA8	95,486	95,449	77,810	81.49
KA1	92,028	92,007	72,285	78.55
KA1	10,488	10,486	83,499	79.61
KA3	91,201	91,177	72,514	79.51
KA4	93,957	93,931	74,117	78.88
KA5	11,338	11,335	92,514	81.6
KA6	94,245	94225	77,331	82.05
KA7	10,717	10,715	87,067	81.24
KA8	11,862	11,860	96,961	81.74
LA1	10,941	10,939	90,034	82.29
LA2	11,996	11,992	95,986	80.01
LA3	10,820	10,817	85,336	78.87
LA4	11,715	11,712	93,585	79.88
