# Supplementary figures and images for: Oral Administration of Lactobacillus amylovorus Alleviates Diarrhea by Restoring Gut Microbiota and SCFAs in Neonatal Goats
Source: Animals (Basel). 2026 Feb 16;16(4):633. doi: 10.3390/ani16040633 (PMC12937376; doi:10.3390/ani16040633)

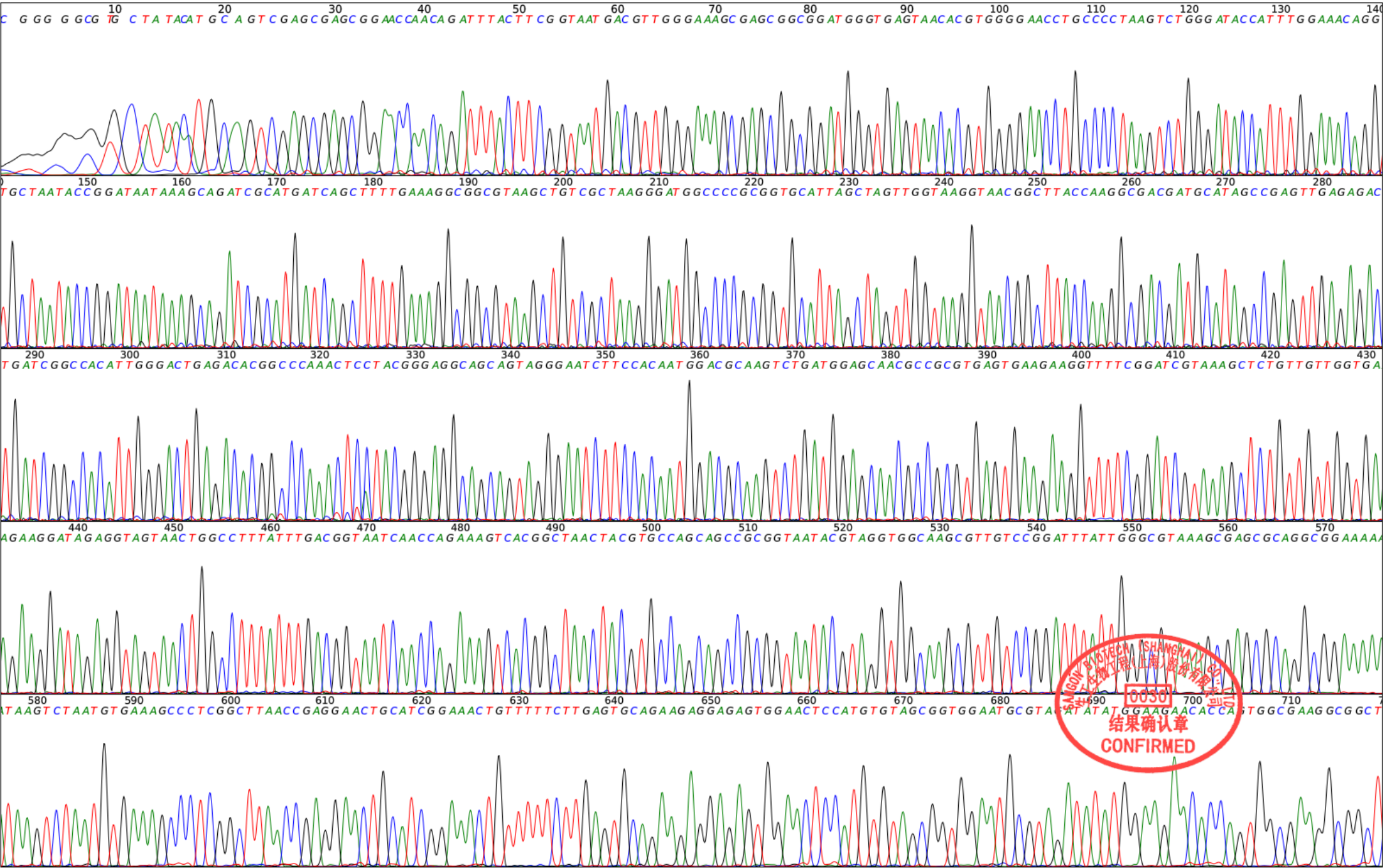

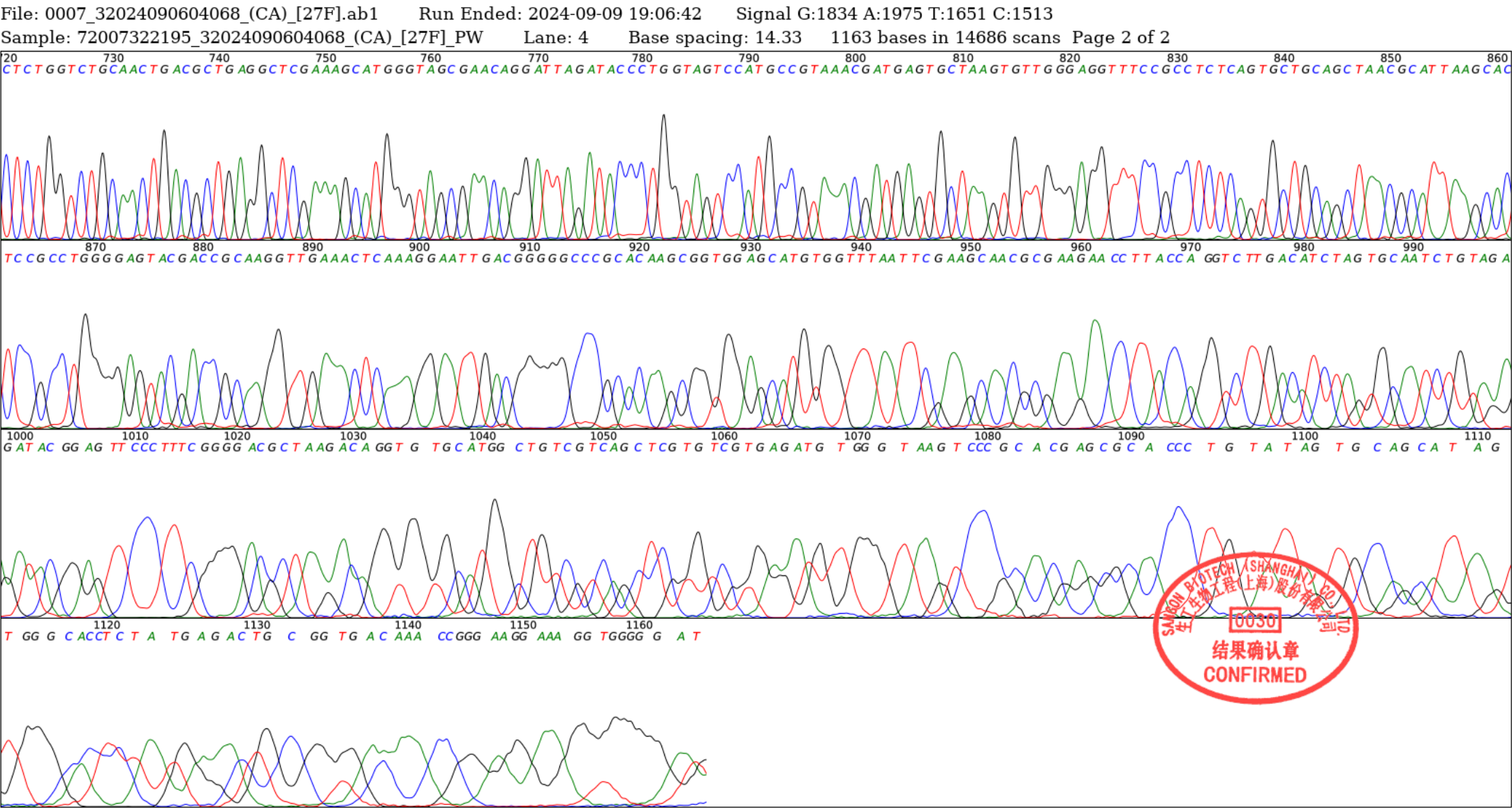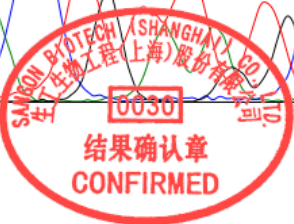

Supplement: Supplementary file 1 [file animals-16-00633-s001.zip › 0007_32024090604068_(CA)_[27F]_H.pdf]

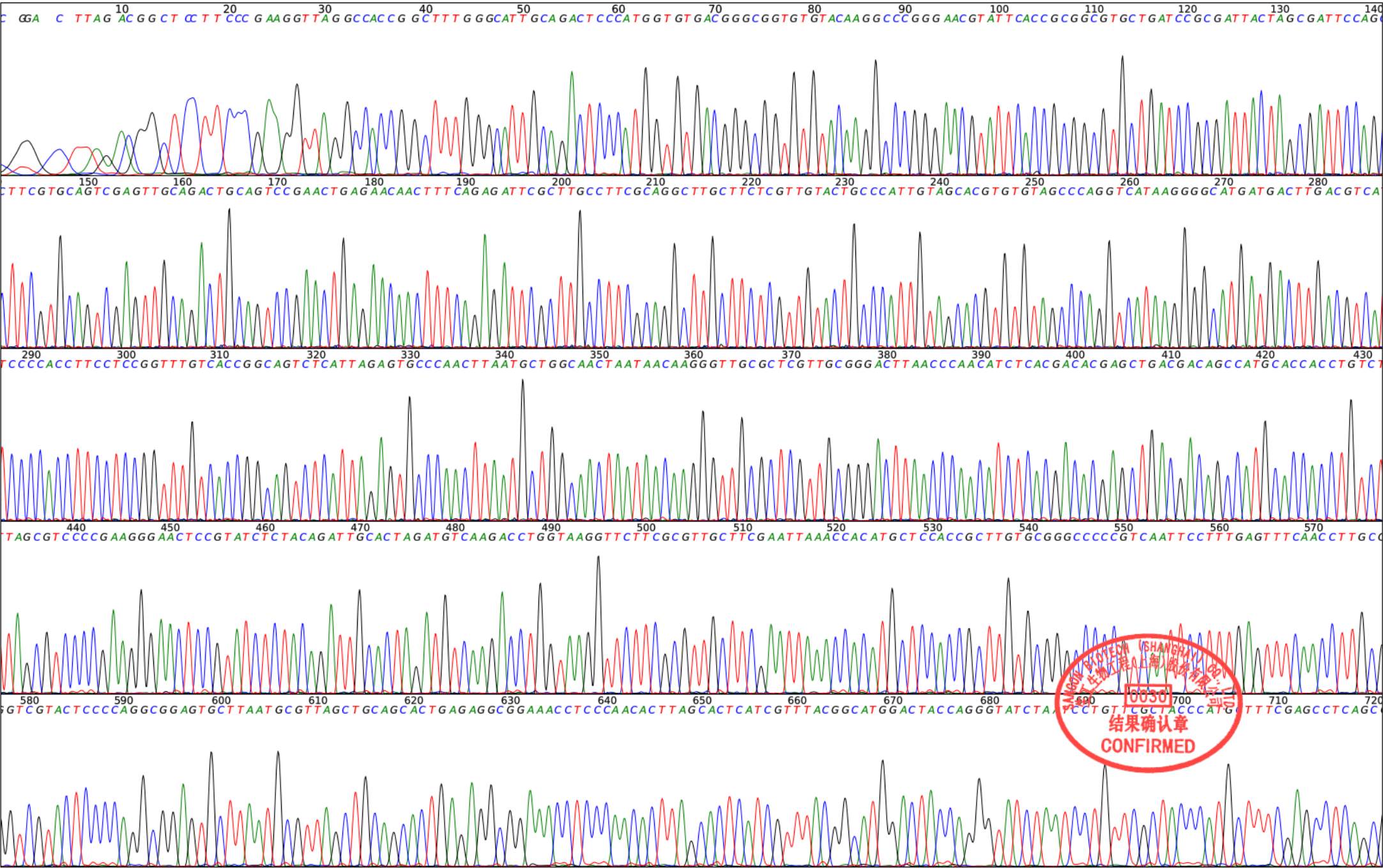

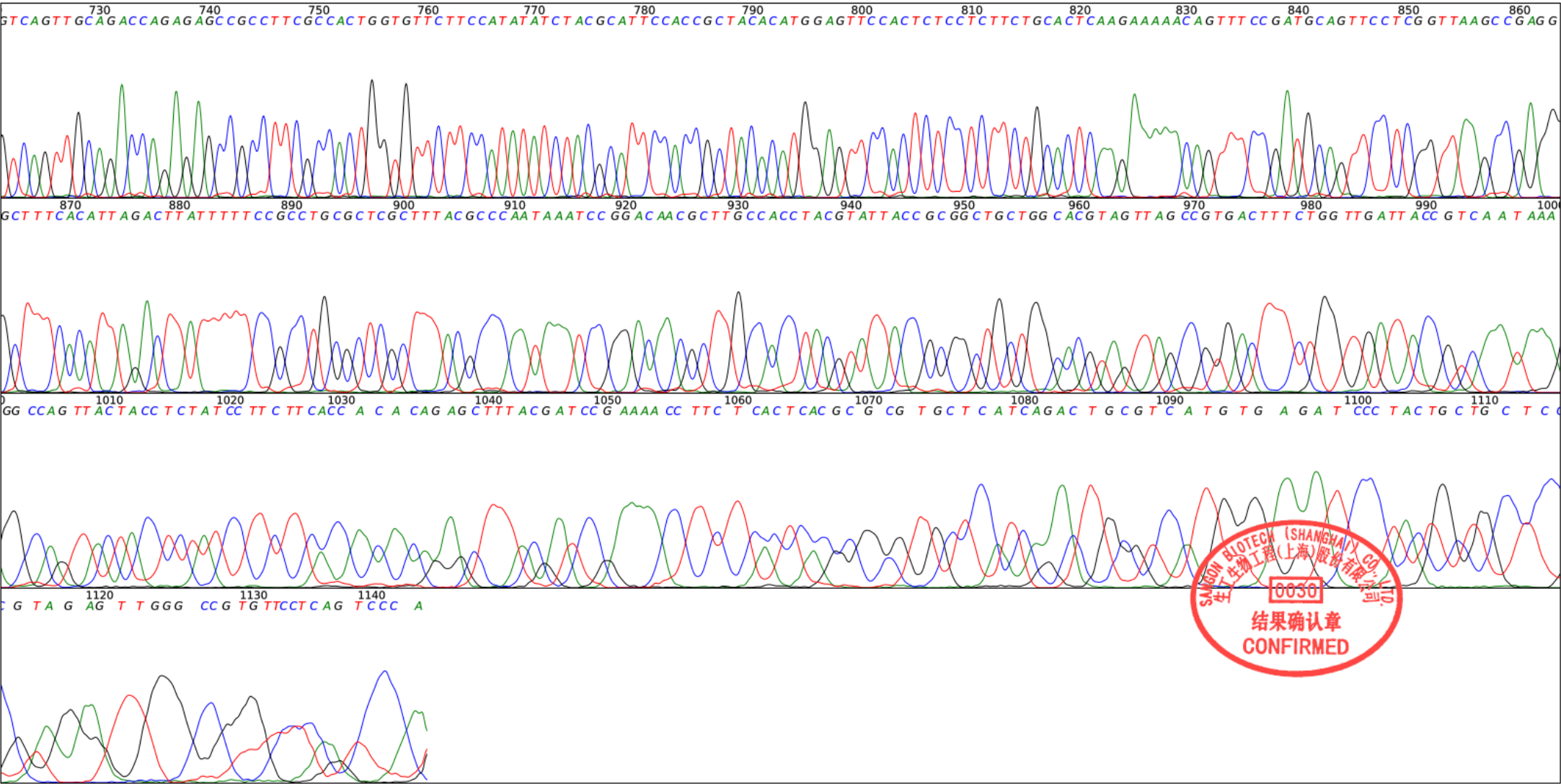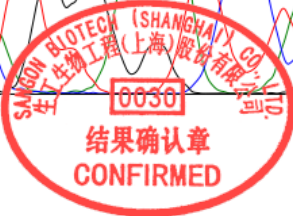

Supplement: Supplementary file 1 [file animals-16-00633-s001.zip › 0008_32024090604068_(CA)_[1492R]_H.pdf]
